# Influence of Oxidative Stress Generated by Smoking during Pregnancy on Glutathione Status in Mother-Newborn Pairs

**DOI:** 10.3390/antiox10121866

**Published:** 2021-11-24

**Authors:** Magdalena Chełchowska, Joanna Gajewska, Jadwiga Ambroszkiewicz, Joanna Mazur, Mariusz Ołtarzewski, Tomasz M. Maciejewski

**Affiliations:** 1 Department of Screening Tests and Metabolic Diagnostics, Institute of Mother and Child, Kasprzaka 17a, 01-211 Warsaw, Poland; joanna.gajewska@imid.med.pl (J.G.); jadwiga.ambroszkiewicz@imid.med.pl (J.A.); mariusz.oltarzewski@imid.med.pl (M.O.); 2 Department of Humanization in Medicine and Sexology, Collegium Medicum University of Zielona Góra, 65-729 Zielona Góra, Poland; j.mazur@cm.uz.zgora.pl; 3 Clinic of Obstetrics and Gynaecology, Institute of Mother and Child, Kasprzaka 17a, 01-211 Warsaw, Poland; dyrektor.naczelny@imid.med.pl

**Keywords:** smoking in pregnancy, cord blood, glutathione, glutathione peroxidase, glutathione reductase, total oxidant capacity

## Abstract

Glutathione plays a key role in maintaining a physiological balance between prooxidants and antioxidants in the human body. Therefore, we examined the influence of maternal smoking as a source of oxidative stress measured by total oxidant capacity (TOC) on reduced glutathione (GSH), oxidized glutathione (GSSG), glutathione peroxidase (GPx-3), and reductase (GR) amount in maternal and umbilical cord blood in 110 (45 smoking and 65 non-smoking) mother-newborn pairs. Concentrations of glutathione status markers and TOC were evaluated by competitive inhibition enzyme immunoassay technique. Plasma TOC levels were significantly higher and the GSH/GSSG ratio, which is considered an index of the cell’s redox status, were significantly lower in smoking women and their offspring than in non-smoking pairs. Decreased GR levels were found in smoking mothers and their newborns compared with similar non-smoking groups. Although plasma GPx-3 concentrations were similar in both maternal groups, in the cord blood of newborns exposed to tobacco smoke in utero they were reduced compared with the levels observed in children of tobacco abstinent mothers. Oxidative stress generated by tobacco smoke impairs glutathione homeostasis in both the mother and the newborn. The severity of oxidative processes in the mother co-existing with the reduced potential of antioxidant systems may have a negative effect on the oxidative-antioxidant balance in the newborn.

## 1. Introduction

Metabolic disorders, including disorders of reduction-oxidation (redox) processes, are important for the etiology and development of reproductive, pregnancy, fetal, and newborn diseases [1,2,3,4]. Free radical (FR) damage can be a common denominator for many biochemical processes, including inflammatory, autoimmune, and neurotoxic [5,6].

Pregnancy is defined as a state of moderate oxidative stress associated with the intensification of metabolic processes and an increased demand for oxygen in the maternal-fetal compartment [1]. Simultaneous smoking of cigarettes and thus exposure to tobacco smoke in utero causes an additional increase in free radical processes in both the mother and the developing fetus [7]. Tobacco smoke is an important source of many FRs from both the gas phase and the tar fraction. Semiquinone (QH^•^), quinone (Q), and hydroquinone (QH_2_) radicals predominate in the tar fraction of smoke, while hydroxyl radical (^•^OH), superoxide anion (O_2_^•−^), and hydroperoxide radical (HO_2_^•^) dominate in the gas phase. There is also hydrogen peroxide (H_2_O_2_) in cigarette smoke, which is a precursor to the formation of a reactive ^•^OH, and nitric oxide (NO), which can become a source of the toxic peroxynitrite (ONOO^−^) [8].

In the case of an uncomplicated pregnancy, the intensification of free radical processes is under the control of the antioxidant response, which protects cells against the adverse effects of oxidative stress. [1]. In contrast, exposure to tobacco smoke during pregnancy may impair the defense activity of both antioxidant enzymes and small-molecule non-enzymatic antioxidants [9,10,11]. We have previous evidence that oxidative marker levels (e.g., TOC—total oxidant capacity, MDA—malondialdehyde, ox-LDL—oxidized low-density lipoprotein) were higher and antioxidant concentrations (e.g., TAC—total antioxidant capacity, vitamins A, E, and adiponectin) were lower in smoking pregnant women than in non-smoking ones [12,13].

One of the most effective non-enzymatic antioxidants necessary to maintain the oxidation-reduction balance is glutathione (GSH). This tripeptide (γ-glutamyl-cysteinyl-glycine) is the most ubiquitous low molecular weight water-soluble thiol compound found in all prokaryotic and eukaryotic cells at millimolar concentrations (1–10 mM) [14]. In a typical eukaryotic cell, 80–85% of glutathione is located in the cytosol, which is the main synthesis site of this compound; and the remainder is found in the mitochondria, nucleus, and endoplasmic reticulum (about 10–15%) [15]. The main antioxidant role of glutathione results from the possibility of reducing endogenous H_2_O_2_ to water and O_2_, and converting organic hydroperoxides to their corresponding alcohols. During the hydrogen peroxide scavenging process, GSH is oxidized to glutathione disulfide GSSG by glutathione peroxidase (GPx), which catalyzes the reaction. Regeneration of the active form of GSH takes place as a result of enzymatic reduction of GSSG via NADPH-dependent glutathione reductase (GR). GR plays an important role in the glutathione redox cycle, maintaining a balance in the GSH/GSSG ratio, which is crucial for cellular activity and survival. Reduced glutathione also directly reacts non-enzymatically with different types of FRs, e.g., NO, O_2_^•−^ and HO_2_^•^, and regenerates other antioxidants, e.g., α-tocopherol and ascorbic acid [7,16,17].

The role of glutathione in the body is not limited to the removal of free radicals. This protein is involved in the processes of xenobiotic detoxification, redox-dependent cell signaling regulation, DNA and protein synthesis, cell differentiation, proliferation and apoptosis, and immune system modulation [14,17]. Additionally, through the γ-glutamyl cycle, GSH is a constant source of cysteine. In the glutathionylation process, GSH keeps the thiol groups of cysteine-rich proteins in a reduced state, which ensures their functional activity [18].

There are limited reports on the effects of active smoking on the glutathione profile in pregnant women and their offspring, and the results are inconclusive [19,20,21,22]. In vitro studies on the influence of tobacco smoke on GSH and its oxidate form GSSG, carried out using different human cell models, showed that glutathione protects cells against cigarette smoke-induced oxidative stress [23]. Therefore, we examined the effect of maternal smoking as a source of oxidative stress measured by total oxidant capacity levels on reduced glutathione, oxidized glutathione, glutathione peroxidase 3, and glutathione reductase amounts in maternal and umbilical cord blood. We also assessed the relationship between the studied biochemical parameters and determined the cell redox index (GSH/GSSG) in the mothers and their offspring.

## 2. Materials and Methods

### 2.1. Study Population

The study complies with the ethical standards established in the Declaration of Helsinki and was approved by the Ethics Review Committee of the Institute of Mother and Child in Warsaw, Poland (approval no. 20/2018). All participants were informed about the study goals and procedures and were asked to give their written consent for biological sample analysis and the use of demographic and clinical information collected from the patients’ medical records.

The study was carried out in 110 mother-newborn pairs, who were patients of the Obstetrics and Gynecology Clinic at the Institute of Mother and Child in Warsaw, Poland between March 2018 and December 2019. The presented work is a continuation of research on oxidative stress in smoking pregnant women and their children conducted by the authors in earlier years, therefore, the previously described criteria of selection for the groups were used [24]. All women had uncomplicated singleton pregnancies and normal vaginal birth at term (37–42 weeks of gestation). Mother’s obstetric and internal disturbances (gestational diabetes mellitus, hypertension, preeclampsia, premature delivery, active hepatitis, renal and cardiovascular diseases, delivery complications, and prolonged labor or assisted reproduction) and newborn’s health problems (birth defects found during gestation, weight not appropriate for gestation), as well as maternal restricted diet (vegetarian or diabetic diet), using drugs and drinking alcohol constituted the exclusion criteria.

The study group consisted of a successive series of 45 women who smoked at least five cigarettes a day during the entire pregnancy and smoked for a minimum of two years before getting pregnant, and their offspring. The control group consisted of 65 non-smoking women of a similar age and gestational age and their newborns. Qualification for the smoking group was based on self-reported information about cigarette smoking habits and verified by the determination of cotinine in the blood serum of both the mothers and their children.

Clinical data on the course of pregnancy, gestational age, mode of delivery, condition of the newborn, including sex, birth weight, body length, and the Apgar score, were collected in the days following delivery.

In both groups of women, pre-pregnancy body mass index (BMI) was calculated based on the data obtained from their medical records.

Pre-pregnancy BMI was calculated using the equation: pre-pregnancy weight (kg)/height (m^2^)

The anthropometric parameters of the newborns were evaluated within 24 h after birth. Birth body length and weight were determined using a measuring board to the nearest 0.1 cm and a calibrated scale to the nearest 10 g. The ponderal index (PI) in infants was calculated using the formula [25]:

PI = [weight (grams) × 100]/[height (cm)]^3^ (1)

### 2.2. Blood Collection

Maternal peripheral blood samples (5 mL) were collected on the day of admission for delivery while performing routine examinations. Cord blood samples (5 mL) were obtained from the umbilical vein at the time of delivery after cutting the umbilical cord.

The blood samples were prepared specifically for the biochemical tests. The samples (serum and EDTA plasma) were divided into small portions and immediately frozen at −80 °C until biochemical analyses were performed (stored no longer than two months).

### 2.3. Maternal and Cord Blood Cotinine Concentration

Serum cotinine concentration as a marker of exposure to tobacco smoke was measured using a commercially available kit, Cotinine Direct ELISA (cat. No.: CO096D, Calbiotech Inc., Spring Valley, CA, USA). The intra- and inter- assay coefficient of variation (CV) of this method was less than 10% and the analytical sensitivity limit was 5.0 ng/mL. Based on a population study of pregnant women, we used a cut-off value of ≥13.7 ng/mL to distinguish smokers from non-smokers [26].

### 2.4. Maternal and Cord Blood Oxidative Status Markers

Markers of oxidative stress (GSH, GSSG, GPx-3, GR, TOC) levels in plasma were determined using enzyme-linked immunosorbent assay (ELISA), strictly in line with the manufacturer’s instructions.

Total oxidant capacity values were measured by a colorimetric assay based on the enzymatic reaction of peroxides and peroxidases (cat No. DMP-4200, Omnignostica Forschungs GmbH, Hoflein/Danube, Austria). The intra- and inter-assay coefficients of variation were less than 4.9% and 7.33%, respectively, and the limit of detection was 0.06 mmol/L. The concentrations of the standards are given in H_2_O_2_ equivalents (mmol/L). Plasma peroxide levels were calculated as the difference of the absorbance readings relating to the hydrogen peroxide standard curve [27].

GSH and GSSG concentrations were measured using Human (GSH) ELISA Kit (cat. No.: 201-12-5407) and Human (GSSG) ELISA Kit (cat. No.: 201-12-5444, SunRed Biotechnology Company, Shanghai, China) based on sandwich enzyme-linked immunosorbent assay using two specific and high-affinity monoclonal antibodies. The GSH in the samples bonded to the first antibody specific to glutathione coated on the microtiter plate. In the following step, the second specific anti-GSH antibody bonded in turn to the immobilized GSH. The second antibody was biotinylated and was incubated in a mixture with a streptavidin-peroxidase-enzyme Conjugate. In the closing substrate reaction, the turn of the color was catalyzed quantitatively depending on the GSH level of the samples. The GSSG concentration was determined in the same way using analogous GSSG antibody. The intra- and inter-assay CVs were less than 10% and 12% for GSH, and 8.0% and 11.0% for GSSG, respectively. The sensitivity of the test was 0.118 μmol/L for GSH and 0.045 μmol/L for GSSG, respectively.

The GSH/GSSG ratio, which is considered an index of the cell’s redox, was calculated.

The GPx-3 protein amount was determined using Human GPX3 (Glutathione peroxidase 3) ELISA Kit (cat. No. EH2531, Fine Biotech Co., Ltd., Wuhan, China) based on sandwich ELISA technology. The microplate provided in this kit had been pre-coated with an antibody specific to GPx-3. Standards or samples were added to the appropriate microplate wells with a biotin-conjugated antibody specific to GPx-3. Next, Avidin conjugated to horseradish peroxidase (HRP-streptavidin) was added to each microplate well and incubated. TMB (3,3′,5,5′-tetramethylbenzidine) substrates were used to visualize the HRP enzymatic reaction. The concentration of GPx-3 in the samples was determined by comparing the O.D. of the samples to the standard curve. The GR protein concentration was measured in an analogous manner using Human GR (Glutathione reductase) ELISA Kit (cat. No. EH3170, Fine Biotech Co., Ltd., Wuhan, China). The intra- and inter-assay coefficients of variation were less than 8.0% and 10% for both GPx-3 and GR and the assay sensitivity was less than 0.938 ng/mL for GPx-3 and 46.875 pg/mL for GR, respectively. In order to make the diluted target protein concentration fall in the optimal detection range of the kit, the target protein concentration (GPx-3 or GR) in the test sample was estimated and a 1:2 dilution factor was used.

### 2.5. Statistical Analysis

All statistical analyses were processed using IBM-SPSS software version 23.0 (SPSS INC., Chicago, IL, USA). The Kolmogorov–Smirnov test was used to assess the normality of data distribution for continuous variables. Results were presented as mean values and standard deviation (SD) for symmetrically distributed data or as median values and interquartile range (IQR) for skewed distributions. Differences between the non-smoking and smoking groups of mothers and their neonates regarding anthropometrical and biochemical parameters were evaluated using Student’s *t*-test and the Mann–Whitney *U*-test depending on the assumptions. Categorical data were compared using the chi-squared test. For the maternal and neonatal group comparisons, the Wilcoxon matched-pairs test was used.

For simple correlation analysis, Spearman’s rank-order correlation coefficients were calculated to evaluate the relationships between the studied markers. Multivariate regression models with GSH and GSSG concentrations as the dependent variables were evaluated to examine the potential impact of glutathione enzymes, TOC, and smoking status. The results were presented as both standardized (β regression coefficient) and unstandardized parameters (B regression coefficient with a 95% confidence interval—CI). The R^2^ coefficient of determination was considered as a goodness of fit statistics. The significance level was set at a *p*-value of <0.05.

## 3. Results

### 3.1. Study Population

We examined 110 healthy pregnant women of Caucasian origin and their offspring. Both average age (27.9 ± 4.9 vs. 29.3 ± 4.5 years, *p* = 0.159) and gestational age (39.07 ± 1.0 vs. 39.38 ± 0.9 weeks of gestation, *p* = 0.052) were similar in the groups of smokers and non-smokers, respectively. In addition, the mothers’ baseline anthropometric parameters: pre-gravid BMI (23.3 ± 1.8 vs. 23.6 ± 2.0 kg/m^2^, *p* = 0.633), weight (62.6 ± 5.9 vs. 64.6 ± 6.5 kg, *p* = 0.110), height (1.64 ± 0.04 vs. 1.66 ± 0.05 m, *p* = 0.068) did not differ in smokers and non-smokers, respectively.

The percentage of girls and boys was similar in both groups of newborns, with 47.7%/52.3% in the non-smoking group and 46.7%/53.3% in the smoking one (*p* = 0.535). The average Apgar score in the fifth minute was 10 (range: 9–10 points) and did not differ between the studied groups (*p* = 0.405).

**Figure 1:**
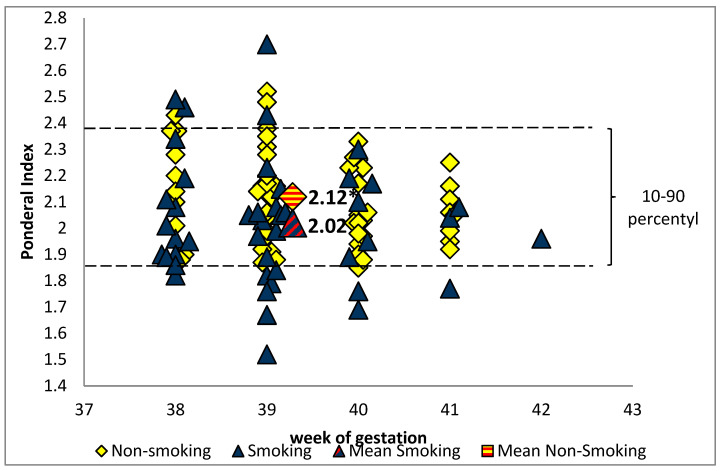
The ponderal index value in the group of newborns of smoking and non-smoking mothers (* *p* < 0.05).

Children of active smokers had a lower birth length (53.5 ± 2.5 cm vs. 54.9 ± 2.4 cm, *p* = 0.012) and birth weight (3096 ± 392 g vs. 3496 ± 378 g, *p* < 0.001) than children of tobacco abstinent mothers. In the smoking group, six newborns (one boy and five girls) had a birth weight of less than 2500 g (range: 2220–2480 g), while in the non-smoking group the lowest birth weight was 2940 g. The mean value of PI for the full-term infants was significantly lower in the smoking than the non-smoking group (*p* = 0.011). The ponderal index below 10 percentile was observed only in the smoking group and affected about 22% of infants (Figure 1).

### 3.2. Maternal and cord Blood Cotinine Concentrations

In the group of smoking mothers, the mean serum concentration of cotinine was 87.9 µg/L (range: 40.1–151.7 µg/L) and strongly correlated (r = 0.881, *p* < 0.001) with cotinine levels in the cord blood of their children (mean: 68.9 µg/L, range: 36.7–124.4 µg/L). In the group of non-smoking mother-newborn pairs, serum cotinine was at a non-detection level. Women smoked an average of nine (range 5–20) cigarettes a day throughout gestation, with 53.3% smoking 5 to 9 cigarettes, 33.4% smoking 10 to 14 cigarettes, and 13.3% smoking 15 to 20 cigarettes a day. The average duration of smoking before conception was 7.9 years (range: 2–15 years). The cotinine concentrations in maternal as well as in cord blood were positively associated (*p* < 0.001) with the number of cigarettes/day (r = 0.918; r = 0.881, respectively).

### 3.3. Maternal and Cord Blood Oxidative Status Markers

**Figure 2:**
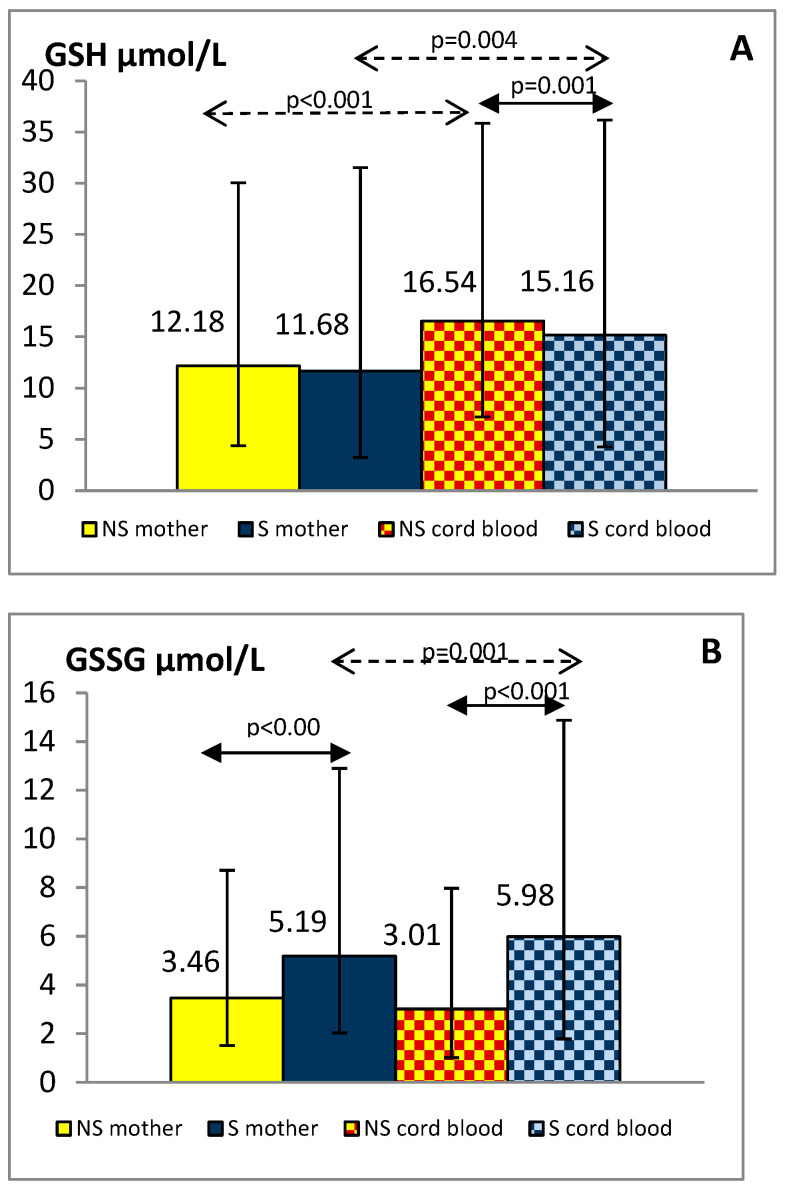

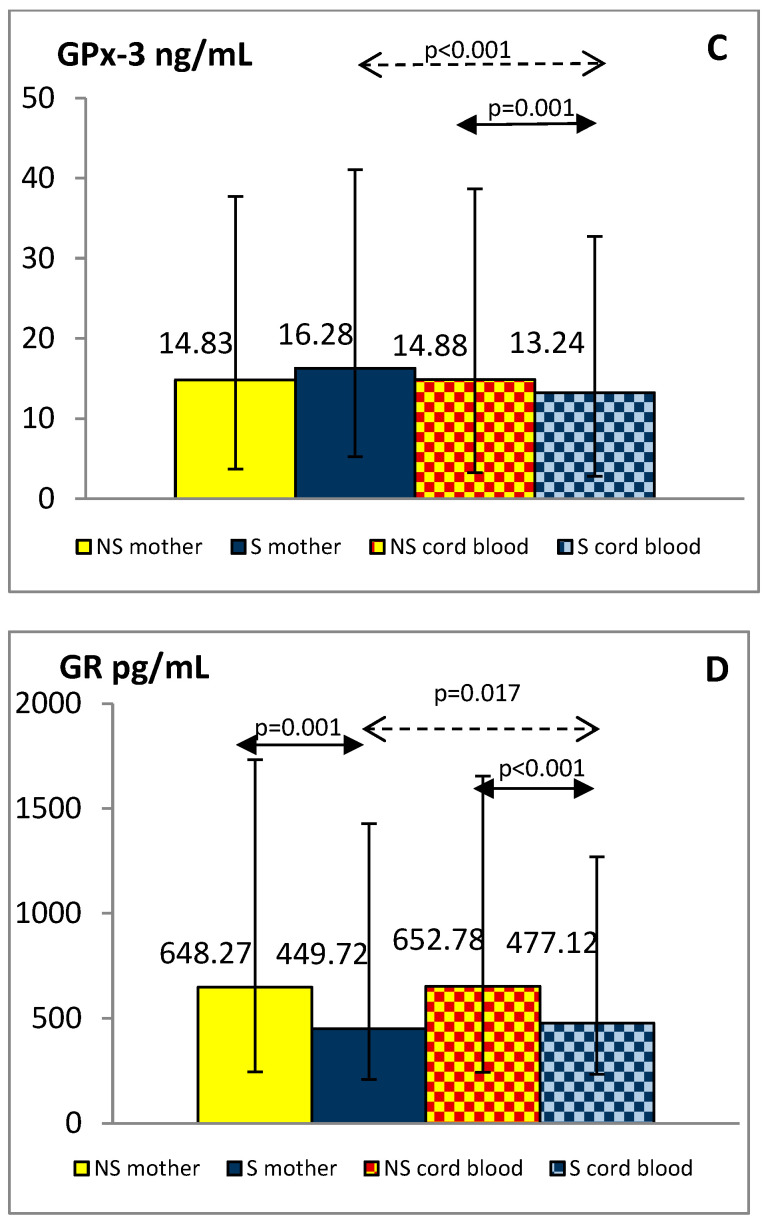
Levels of GSH (**A**), GSSG (**B**), GPx-3 (**C**) and GR (**D**) in the plasma of smoking (S) and non-smoking (NS) mother-newborn pairs. The results are presented as the median and interquartile range (1Q-3Q); GSH—reduced glutathione, GSSG—oxidized glutathione, GPx-3—glutathione peroxidase 3, GR—glutathione reductase.

The plasma concentrations of total oxidant capacity were significantly higher in the smoking group, both mothers and newborns, compared to the group of non-smoking mothers and children (maternal blood: 0.439 ± 0.207 vs. 0.295 ± 0.162 mmol/L, *p* < 0.001; cord blood: 0.322 ± 0.123 vs. 0.183 ± 0.088 mmol/L, *p* < 0.001, respectively). TOC level in the umbilical cord blood was lower by about 27% in the smokers and 38% in the non-smokers group compared with the maternal blood (*p* < 0.001). GSH, GSSG, GPx-3, and GR levels in the blood of mothers and their children are shown in Figure 2A–D.

Plasma GSSG levels were significantly higher in smoking women and their offspring than in non-smoking pairs (*p* < 0.001). Although plasma GSH and GPx-3 concentrations were similar in both maternal groups, in the cord blood of newborns exposed to tobacco smoke in utero they were reduced compared with the levels observed in children of tobacco abstinent mothers (*p* < 0.01). Decreased GR levels were shown in smoking mothers and their newborns against similar non-smoking groups (*p* < 0.01). Additionally, we found lower values of the GSH/GSSG index in smokers than in non-smokers, both mothers and newborns (median: 2.21 vs. 3.83 and 2.19 vs. 5.01, respectively; *p* < 0.001).

In the group of smoking mothers, plasma GSH, GSSG, and GR levels were significantly lower (*p* < 0.01) and GPx-3 significantly higher (*p* < 0.001) than observed in the cord blood of their children. In the non-smoking group, the concentrations of the studied glutathione status parameters did not differ between mothers and newborns except for higher GSH in cord blood (*p* < 0.001).

### 3.4. Relations between GSH, GSSG Levels and Other Biochemical Parameters

The results of Spearman correlation analysis between reduced and oxidized glutathione concentrations and the levels of GPx-3, GR, and cotinine in the groups of non-smoking and smoking mothers and children are presented in Table 1.

Plasma GSH levels were negatively correlated with GSSG, TOC, and positively with GR concentrations in both studied groups of mothers. In addition, negative relations between plasma GSH and GPx-3 as well as cotinine levels were observed in smokers.

In the mother groups, plasma concentrations of GSSG were negatively associated with GR and positively with GPx-3 levels. Moreover, in smokers, higher concentrations of GSSG were also related to increased TOC levels.

In the case of newborns from the group of smoking mothers, a negative relation was found between GSH and TOC as well as cotinine levels, while cord blood concentrations of GSSG were inversely related to GR levels. In the group of the tobacco abstinent, cord blood GSH levels were positively correlated with GR whereas GSSG levels with TOC concentrations.

The results of multivariate regression analyses confirming the detrimental effect of oxidative stress and tobacco smoking during pregnancy on glutathione homeostasis are documented in Table 2 and Table 3.

In the model, estimated for the total group of mothers (smoking and non-smoking women), smoking status, GR and GPx-3 concentrations were important predictors for both GSH and GSSG levels. Additionally, decreased concentrations of reduced glutathione were significantly associated with increased levels of total oxidant capacity in this model.

The analysis performed in the infants group revealed the highest impact of TOC on GSH concentrations whereas smoking status on GSSG levels. Concentrations of GR seem to be an important predictor of both reduced and oxidized glutathione levels in this group.

## 4. Discussion

In pregnant tobacco smokers, maintaining redox homeostasis seems to be particularly important in the context of the well-being of both mother and child [9,10]. Tobacco smoke contains a number of toxic compounds, including oxidants and FRs, which are responsible for the intensification of oxidative stress and the occurrence of pathological changes in the body. A single puff of cigarette smoke includes 10^15^ free radicals in the gas phase and 10^17^ in the tar phase [28]. In the blood of smokers, there are increased levels of products of oxidative damage to proteins, lipids, and DNA as well as decreased activity of antioxidant enzymes and non-enzymatic antioxidants, as a result of which the efficiency of the antioxidant barrier of the organism is impaired [19,21,29].

In the presented study, we observed that the intensification of oxidative processes dependent on tobacco smoke significantly influenced glutathione homeostasis. Moreover, our research confirmed the adverse effect of smoking on the oxidant status of both mothers and children [13,19,20]. As mentioned above, as a thiol compound, glutathione plays an extremely important role in enzymatic and non-enzymatic reactions for protecting cells against oxidative stress damage induced by cigarette smoking [23].

The effect of tobacco smoke on glutathione concentrations has not been clearly established in the general population. Some investigators have not found any change in GSH levels, whereas others have reported increased as well as decreased concentrations of this compound in smokers’ blood compared with non-smokers [28,30,31,32]. However, it has been documented that glutathione levels can be influenced by both the intensity of smoking and the duration of exposure to tobacco smoke [31,33]. Increased blood GSH levels have been shown in heavy smokers (>20 cigarettes a day). On the other hand, in people who smoked less than 20 cigarettes a day, the concentrations of glutathione were similar to those observed in the group of non-smokers [29,31,34].

There are only a few studies on the influence of smoking during pregnancy on maternal and child glutathione status, and the results were inconclusive. Our research has confirmed the results of other authors who showed no differences between the levels of reduced glutathione in the blood of pregnant smokers and non-smokers [20,35]. There are also reports of a negative effect of tobacco smoke on the levels of this compound during gestation [9,21]. However, in one case [21], a study was conducted in smoking pregnant women with intrauterine growth restriction, which may be associated with increased oxygen stress and increased consumption of antioxidant systems. In another study [9], GSH levels were measured in whole blood in a group of active smokers during the first trimester of pregnancy. Gould et al. [33] reported that short-term exposure decreased GSH levels whereas our study was carried out at the end of pregnancy in patients who smoked an average of 7.9 years before conception, indicating long-term exposure to tobacco smoke. Reduced glutathione oxidizes to GSSG as a cofactor for GPx, which catalyzes the H_2_O_2_ scavenging process. To the best of our knowledge, this is the first report to describe the effect of smoke on oxidized glutathione levels in the blood of pregnant women, which in the physiological stage accounts for less than 1–2% of the total GSH pool and increases under conditions of oxidative stress [15,17]. We observed higher levels of GSSG and slightly higher levels of GPx in smokers compared with non-smokers, while other authors showed significantly higher activity of this enzyme [9,19,20]. These differences may be due to the fact that we determined the level of an extracellular form of glutathione peroxidase (GPx-3), which is most abundant in plasma, while others tested total GPx activity [36,37]. We found lower levels of glutathione reductase in the smoking than in the non-smoking group. This may indicate a decreased reduction of GSSG to the active form of GSH and, consequently, may disturb the GSH/GSSG ratio balance and lead to cell damage. The demonstrated positive correlation between GR and GSH levels and the negative correlation with GSSG levels confirm the key role of this enzyme in the glutathione redox cycle.

All newborns, regardless of prenatal tobacco smoke exposure, are particularly susceptible to oxygen damage due to the conditions generating increased production of free radicals with incomplete maturity of antioxidant systems and the high energy demand of rapidly growing tissues [16]. Elevated levels of oxidative stress markers in the cord blood of infants born to mothers who smoked actively during pregnancy were also documented, suggesting that exposure to tobacco smoke in utero intensifies oxidative stress in newborns [13]. Here, we have found a close relationship between TOC concentration, cotinine, and glutathione level in the cord blood. For the first time, we confirmed that maternal smoking increases the concentration of oxidized glutathione and reduces the levels of glutathione reductase in the offspring. Some authors postulated that lower fetal glutathione levels in pregnant smokers may be the result of reduced placental glutathione uptake in this group [38]. In agreement with Bizoń et al. [21] and Aydogan et al. [19], we observed lower levels of GSH and GPx in the cord blood of smokers, while Pizent et al. [10] obtained similar activity of this enzyme in both smoking and non-smoking groups. Interestingly, these authors demonstrated a significant negative effect of smoking on the level of selenium (Se), which is essential for GPx bioactivity [21,36,39]. In our study increased total oxidant capacity with a simultaneous reduction of GSH, GSH/GSSG, GPx, and GR levels in the plasma of the smoker’s newborns may suggest lower adaptation possibilities of blood in protection against oxygen stress in this group.

There are two basic processes influencing the increase in GSH concentration, it is the regeneration of GSH from GSSG and the de novo synthesis of this compound. GSH synthesis in the cell is limited mainly by the availability of its amino acids, especially the reduced form of cysteine [23]. In human blood, this amino acid most often occurs in the oxidized form, cystine, and its availability in the reduced form necessary for GSH neosynthesis is increased by other antioxidants (e.g., ascorbic acid, vitamin E, lipoic acid) [30]. The elevated cystine and decreased cysteine levels observed in smokers may result from a deficiency of the above-mentioned antioxidants in this group [28,30,32,40]. In addition, in vitro cellular model studies have shown that exposure to cigarette smoke extract can induce antioxidant signaling pathways through nuclear factor erythroid-2-related factor-2 (Nrf2). This factor is involved in the regulation of the transcription of many antioxidant genes, including glutamate cysteine ligase (GCL), the rate-limiting enzyme in the synthesis of GSH [41,42]. Finally, the detoxification of large amounts of xenobiotics in tobacco smoke by glutathione through the formation of S-conjugates may lead to a decrease in the concentration of this antioxidant in the blood of smokers [43].

### Strengths and Limitations

The main strength of the presented study was the performance of biochemical measurements in the blood of healthy women actively smoking in pregnancy and in the umbilical cord blood of their offspring. About 50% of them were smoking over 10 cigarettes a day and the remaining over five cigarettes a day throughout gestation. Importantly, the qualification of patients to the groups was confirmed by quantifying cotinine in the blood of mothers and neonates. Another advantage was the comprehensive determination of the concentrations of both forms of glutathione and two key enzymes conditioning glutathione transformation and guaranteeing its antioxidant properties.

The presented study has several potential limitations. The authors are aware of the fact that the analysis results would be strengthened if the study group was larger. However, we believe that because of the homogeneous and unified nature of the group in terms of major factors affecting glutathione levels, even though the study group is not very large, the results are significant [44]. Another limitation of the study is that we did not measure other important antioxidants involved in glutathione homeostasis (e.g., Nrf2, thioredoxin, and glutaredoxin family enzymes), but we are planning to examine these relationships in future studies [41]. Moreover, we used plasma as a research material whereas whole blood or red blood cells lysates are responsible for 10% of total glutathione synthesis in humans [14]. Measuring the concentrations of glutathione parameters in various biological materials allows for the assessment of its differential effect on the pregnant woman and the developing fetus.

## 5. Conclusions

Oxidative stress generated by the toxic compounds in cigarette smoke impairs the homeostasis of glutathione in both mother and newborn. The severity of oxidative processes in the mother coexisting with the reduced potential of antioxidant systems may have a negative impact on the oxidative–antioxidant balance in the newborn. This can be confirmed by the twice lower value of the cell’s redox status index expressed by the GSH/GSSG ratio in neonates of smoking mothers compared with the non-smoking group.

## Figures and Tables

**Table 1:** Spearman’s rank correlation coefficients between GSH as well as GSSG and other studied biochemical parameters in non-smoking and smoking mother-newborn pairs.

Biochemical Parameters	GSH (μmol/L)				GSSG (μmol/L)			

	Mothers		Newborns		Mothers		Newborns	
	Rho	*p*-Value	Rho	*p*-Value	Rho	*p*-Value	Rho	*p*-Value
Non-smoking Group								
GSH (μmol/L)	-	-	-	-	−0.423	<0.001	0.143	0.257
GSSG (μmol/L)	−0.423	<0.001	0.143	0.257	-	-	-	-
GPx-3 (ng/mL)	−0.204	0.102	−0.049	0.701	0.267	0.032	0.073	0.562
GR (pg/mL)	0.446	<0.001	0.317	0.010	−0.376	0.002	−0.228	0.067
TOC (mmol/L)	−0.434	<0.001	−0.110	0.383	0.373	0.002	0.258	0.038
Smoking Group								
GSH (μmol/L)	-	-	-	-	−0.309	0.039	0.137	0.370
GSSG (μmol/L)	−0.309	0.039	0.137	0.370	-	-	-	-
GPx-3 (ng/mL)	−0.510	<0.001	−0.173	0.255	0.407	0.006	0.248	0.101
GR (pg/mL)	0.408	0.005	0.229	0.134	−0.498	0.001	−0.378	0.011
TOC (mmol/L)	−0.442	0.002	−0.573	<0.001	0.087	0.568	−0.058	0.705
Cotinine (μg/L)	−0.480	0.001	−0.532	<0.001	0.275	0.068	0.187	0.219

GSH—reduced glutathione, GSSG—oxidized glutathione, GPx-3—glutathione peroxidase 3, GR—glutathione reductase, TOC—total oxidant capacity.

**Table 2:** Multivariate regression model for GSH in pregnant women and umbilical cord blood.

	Mothers *n* = 110				Newborns *n* = 110			
Variable	B	95%CI	β	*p*-Value	B	95%CI	β	*p*-Value
Smoking status No = 0, Yes = 1	−2.939	−4.376/−1.502	−0.383	<0.001	1.211	−1.019/3.441	0.158	0.284
GSSG	−0.192	−0.653/0.270	−0.089	0.412	0.626	0.124/1.128	0.332	0.015
GPx-3	−0.150	−0.274/−0.025	−0.196	0.019	−0.051	−0.218/0.117	−0.055	0.549
GR	0.005	0.002/0.007	0.354	<0.001	0.005	0.002/0.008	0.301	0.005
TOC	−6.716	−10.026/−3.405	−0.345	<0.001	−9.527	−15.995/−3.060	−0.313	0.004
R^2^ (%)	36.3				19.8			

β—standardized regression coefficient, B—unstandardized regression coefficient, CI—confidence interval, GSH—reduced glutathione, GSSG—oxidized glutathione, GPx-3—glutathione peroxidase 3, GR—glutathione reductase, TOC—total oxidant capacity, smoking status was coded as 0 for non-smoking and 1 for smoking mothers.

**Table 3:** Multivariate regression model for GSSG in pregnant women and umbilical cord blood.

Variable	Mothers *n* = 110				Newborns *n* = 110			
	B	95%CI	β	*p*-Value	B	95%CI	β	*p*-Value
Smoking status No = 0, Yes = 1	−1.518	−2.097/−0.940	−0.428	<0.001	−2.777	−3.429/−2.125	−0.683	<0.001
GSH	−0.034	−0.115/0.048	−0.073	0.412	0.089	0.018/0.161	0.169	0.015
GPx-3	0.079	0.027/0.130	0.223	0.003	0.041	−0.022/0.104	0.084	0.201
GR	−0.002	−0.003/−0.001	−0.260	0.002	−0.002	−0.003/−0.001	−0.211	0.006
TOC	0.877	−0.608/2.361	0.097	0.244	0.738	−1.803/3.280	0.046	0.566
R^2^ (%)	47.6				59.2			

β—standardized regression coefficient, B—unstandardized regression coefficient, CI—confidence interval, GSH—reduced glutathione, GSSG—oxidized glutathione, GPx-3—glutathione peroxidase 3, GR—glutathione reductase, TOC—total oxidant capacity, smoking status was coded as 0 for non-smoking and 1 for smoking mothers.

## Data Availability

The data is contained within the article.
